# A qualitative systematic review of the implications of desexed language in women’s healthcare and healthcare literature

**DOI:** 10.1177/17455057261430199

**Published:** 2026-03-27

**Authors:** Abigail Greenfield, Ciara Higley, Naomi Black, Majel McGranahan

**Affiliations:** 1 Warwick Medical School, The University of Warwick, Coventry, UK

**Keywords:** health communication, gender identity, mothers, pregnancy, transgender persons, patient-centred care, health services for transgender persons, women, maternity

## Abstract

**Introduction:**

Increasing numbers of people identifying as transgender and gender diverse (TGD) have introduced challenges regarding language used in women’s healthcare. TGD individuals are defined as those whose gender identity does not align with their sex. This growing patient group has ignited debates over whether language in women’s health should be desexed to accommodate TGD individuals, for example, replacing “mother” with “pregnant people.” Some argue such language is inclusive, while others are concerned it is inaccurate and disrespectful.

**Objective:**

To examine the implications of desexed language in women’s healthcare and healthcare literature.

**Design:**

Qualitative systematic review synthesising primary research on desexed language in women’s healthcare.

**Data sources and methods:**

Qualitative studies examining desexed language in women’s healthcare, published 2010–2024, were included. A systematic search was conducted across MEDLINE, PsycINFO, and CINAHL. Risk of bias was assessed using the Critical Appraisal Skills Programme checklist and data analysed thematically.

**Results:**

Six studies were included, with 80 participants, 13 of whom were women who were not TGD. Themes identified: (1) Language and communication – reflecting differing views on desexed language and the importance of consistent definitions of sex and gender identity; (2) Education – gaps in healthcare professionals’ confidence highlight the need for improved clinical education on TGD language use; (3) Structural challenges – issues with clinical documentation and environments; and (4) Barriers to care – poor healthcare experiences meaning TGD patients may avoid seeking care.

**Conclusion:**

This review highlights the need for resources tailored to TGD individuals, clinician education on personalised language use and improvements to clinical documentation to ensure sex is always recorded, with gender identity included if relevant. Insufficient evidence exists to support universal implementation of desexed language in women’s healthcare. Findings suggest it is not well understood or accepted by women who are not TGD and research is needed to understand its impact on wider groups.

## Introduction

Inconsistencies exist across various medical fields on the definitions of sex, gender, and gender-identity.^1^ However, on 16 April 16 2025, the U.K. Supreme Court ruled that references to “sex” in the Equality Act 2010 denote biological sex. Under this judgement a “woman” is defined as a biological female, irrespective of gender identity. This ruling has wide-reaching implications, including for the preservation of single-sex facilities within healthcare and other public services. For the purposes of this review, “sex,” “female,” and “women” are used to refer to biological sex.

Sex is a reproductive categorisation based on whether individuals produce or are on a developmental pathway to produce, small motile gametes (male) or large sessile gametes (female).^2^ “Gender” reflects the societal and cultural expectations associated with each sex which creates hierarchical social structures affecting males and females differently.^2^ “Gender identity” is often described as an “internal sense of one’s gender,” as a man, a woman, both, or neither. However, there is not a single accepted meaning and definitions are often circular. Some individuals have a sense of gender identity that they experience as being in conflict with their sex and are transgender and gender diverse (TGD), whereas others deny they have a gender identity.^3^

The last decade has seen an increase in individuals identifying as transgender or non-binary.^4,5^ There is a growing number of gender identities and definitions vary widely across the literature.^3^ Non-binary is an umbrella term for people who reject the gender attributions associated with the sexes. Some believe they may have elements of both genders; some may move fluidly between genders (gender-fluid) and others may view themselves as neither male nor female.^6^ For the purposes of this review, those with gender identities differing from their sex will be referred to as TGD individuals.

Some individuals, recorded as female at birth, undertake medical interventions including testosterone hormonal treatment or chest masculinisation mastectomy. However, their sex remains relevant to their health needs, and some may need to access specific women’s healthcare services.

Women’s healthcare refers to sex-specific healthcare services encompassing sexual, reproductive and gynaecological health. To accommodate for the experience of TGD people, there is a growing movement to use desexed language in women’s healthcare. These terms obscure references to sex, for example, replacing “mother” with “birthing people” and “parents,” or substituting “breastfeeding” with “human milk feeding,” “lactation,” or “chest feeding.” Similarly, expressions such as “menstruators,” “cervix owners,” or “people with vaginas” are used in the place of “women.” These changes extend to healthcare literature including articles, textbooks, curricular and organisational documents. Some argue that adopting desexed language promotes inclusivity, labelling it as “gender-inclusive language.”^7^ However, others label desexed language as exclusionary as it serves to centre TGD people as the primary audience. This may inadvertently exclude large numbers of readers such as those who do not understand the concept of gender identity due to cultural or linguistic differences, those with lower literacy, or those who object to being described with desexed terms.^8^

Inclusivity in healthcare communication is understood to mean providing information that is clear, accessible, and useable for the widest audience possible.^9^ This involves maximising accessibility for those with a lower literacy level through communicating in plain language. Plain language involves removing technical jargon, choosing everyday words, presenting information in a clear order, and keeping messages concise so that readers can understand and apply them immediately.^9^ Bartick et al.^8^ suggested that words such as “women” and “mothers” align best with the principles of clear, plain language communication in women’s healthcare.

Moving to desexed language in women’s healthcare was challenged by Gribble et al.,^10^ who raised concerns about desexed language in relation to maternity care. They asserted that it will paradoxically reduce overall inclusivity for women, particularly those less familiar with physiological terms such as “cervix.” This includes women with learning difficulties or those for whom English is not their first language. They argue that gender-inclusive language is “culturally imperialist,” imposing Western notions of gender-identity, therefore is regressive not progressive. Terms like “birth-giver” reduce women to body parts and “chest” conflates “breasts” with the heart and thorax, introducing inaccuracies. Gribble et al. argued “additive language” (e.g., “mothers and parents”) risks inappropriately including fathers and extended family, reducing the visibility of the mothers’ unique health needs. As an example, Bartick et al.^8^ noted that phrases such as “women and birthing people” can shift the meaning of “women” and “mothers” from referring to people of the female sex to referring to gender identities. Gribble et al. proposed targeted resources for TGD people to address their individual needs, highlighting the importance of consistently defining sex and gender-identity.^10^

Pezaro et al.^11^ opposed the arguments described by Gribble et al., proposing a guide for the use of desexed language in midwifery services. Pezaro et al. understood sexed language to be both “cis-heteronormative” and “colonialist” in its disregard for gender diversity. They drew on historical examples of indigenous populations exhibiting TGD family dynamics, emphasising the importance of community and extended family in childbearing experiences. Pezaro et al. argued that, to achieve reproductive justice, no single birthing group should be prioritised over another. They rejected the use of additive language entirely, arguing that sexed language creates health barriers for TGD people and that making sex invisible in such language would reduce sexism. Pezaro et al. considered terms such as “pregnant people” to be optimally inclusive. Morrison et al.^12^ argued there was risks of maleficence and stigma associated with sexed language for TGD people that may delay care or lead to adverse outcomes.

Discussions on desexed language provoke fierce debate, as illustrated by J.K. Rowling’s 2020 tweet critiquing the term “people who menstruate,” which sparked over 25,000 responses, including both supporters and critics, highlighting the controversy.^13^ National guidance reflects these complexities. “Talking About People,”^14^ a style guide published by the U.K. National Institute for Health and Care Excellence (NICE), provides examples of desexed language, suggesting replacing “offer hormonal treatment to women with endometriosis” with “offer hormonal treatment if there is endometriosis.” Likis et al.^7^ warned making “endometriosis” the subject decentralises women thus steering away from a patient-centred approach. Bartick et al.^8^ argued that this leaves the subject ill-defined, reducing clarity in conveying health information and in patient-directed materials may result in women “missing the message.” This demonstrates how evidence-based clinical practice guidelines are impacted by the controversy.^15^

Bartick et al.^8^ provided examples of the use of desexed language in health communication. Desexed language was adopted by a range of high-profile health and academic institution, including the U.S. Centers for Disease Control and Prevention, *Journal of Human Lactation, The Lancet*, and *The American Academy of Paediatrics*. Phrases used include “bodies with vaginas,” “menstruators,” and “birthing bodies.” Gribble^16^ reported that the use of “pregnant people” and “birthing people” on Google Scholar increased 14-fold and 52-fold, respectively, between 2018 and 2023.

Despite the controversy surrounding desexed language, no previous systematic reviews could be identified which examine the impact of desexed language in women’s healthcare. This qualitative systematic review aims to examine the impact of desexed language in women’s healthcare, exploring service users’ and stakeholders’ perspectives. Through analysis of relevant literature, this review seeks to offer an impartial overview of the evidence on this often-polarised topic.

## Methods

This review followed the PRISMA 2020 checklist.^17^

### Selection criteria

All English-language, peer-reviewed qualitative studies published between 2010 and 2024 that focused on the use of desexed language in women’s healthcare and healthcare literature were included.

### Search

Medline, PsycINFO, and CINAHL were searched from 2010 up to 30 October 2024. A two-layered search strategy was devised (Appendix A): the first layer focused on topics related to women’s healthcare (e.g., “obstetrics and gynaecology,” “women’s health,” “sexual health,” “maternity”) and the second on desexed language (e.g., “gender-neutral language,” “inclusive terminology,” “birth-givers,” “chest-feeders”). The full inclusion and exclusion criteria are presented in Table 1.

**Table 1.** Inclusion and exclusion criteria.

Inclusion criteria	Exclusion criteria
Studies published between 2010 and 2024.	Studies published before 2010.
Qualitative primary research.	Quantitative primary research, literature reviews, systematic reviews, opinion articles, letters or conference abstracts.
Studies directly related to women’s healthcare (obstetric, gynaecological, sexual and reproductive health) and/or healthcare literature (academic journals, guidelines, leaflets, online resources).	Studies not related to women’s healthcare and/or healthcare literature.
Studies directly related to the use of desexed language.	Studies not related to the use of desexed language.

### Selection of articles

Two reviewers conducted independent screening of all titles and abstracts with the aid of the Rayyan^18^ systematic review screening software. The first reviewer then performed a full-text screening of the identified studies, with a second reviewer screening a sample (25%) of the full texts. Any disagreements or uncertainties about the proposed included studies were discussed and resolved, with input from another reviewer when required. There was 100% inter-rater reliability in the full-text screening process.

Some studies used aggregate terms such as “sexual and gender minority.” In this context, “sexual minority” refers to participants whose sexual orientation is towards the same sex, both sexes, or neither sex (asexual), while “gender minority” is synonymous with TGD participants. For this review, only studies that provided sufficient focus on TGD populations were included, and only if TGD-specific data could be clearly extracted and analysed separately.

### Data extraction and analysis

A pre-designed data extraction template was used to extract data into Excel by a single reviewer. Data were extracted on the following: first author; date of publication; study type; journal source; study aims and objectives; participants; country and study design and methods. Data were thematically analysed.^19^ This process involved data familiarisation, code formulation, and theme generation.

### Quality assessment

The Critical Appraisal Skills Programme (CASP) checklist^21^ was used to evaluate the strengths and limitations of the included studies. This was performed in duplicate for a third of the included studies, with conflicts resolved through discussion.

The protocol was pre-agreed; however, the systematic review was not registered publicly as the lead author was a student at the time and so was not permitted to register the review publicly on PROSPERO.

## Results

**Figure 1.**
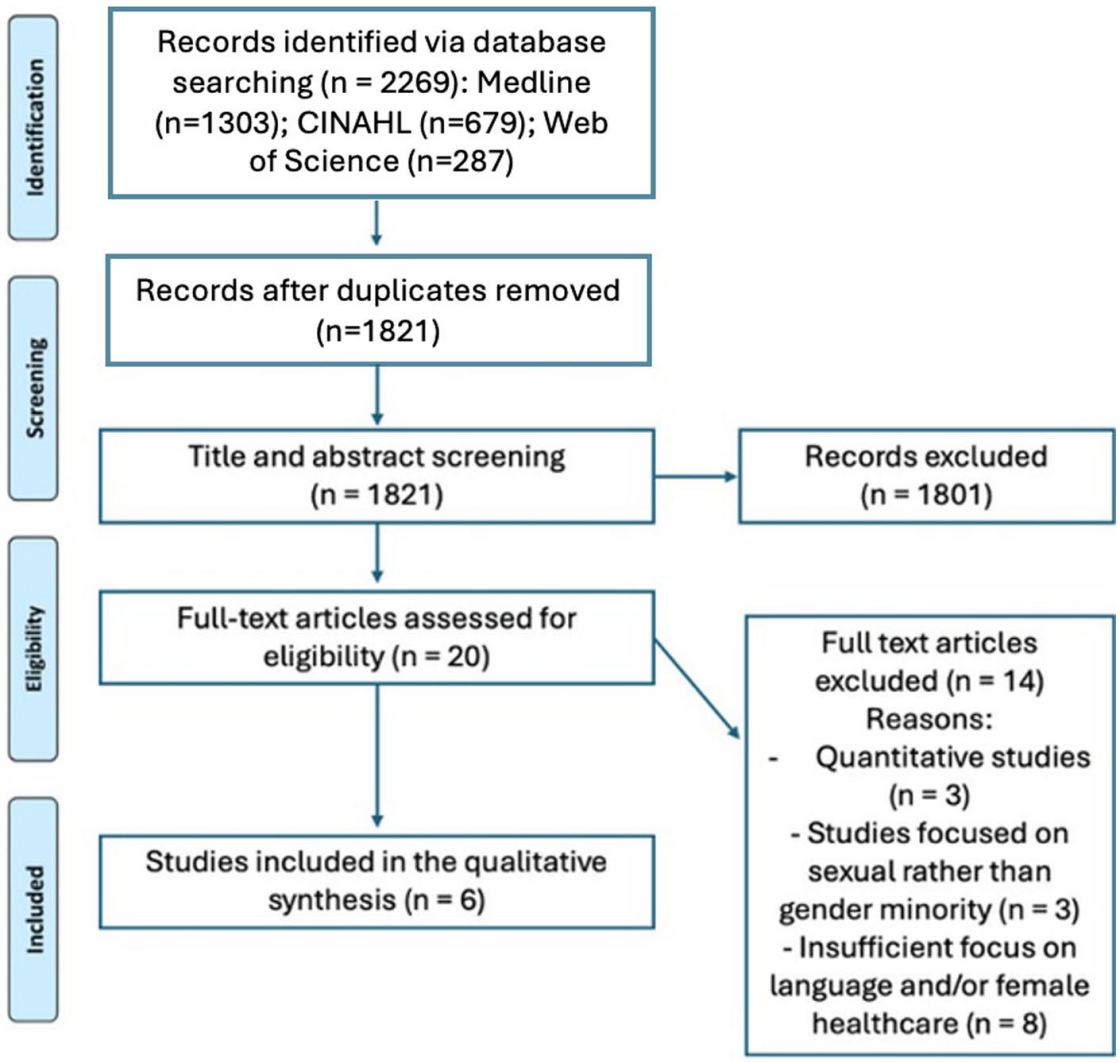
PRISMA flowchart.

In total, 2269 publications were screened and 1821 remained after duplicates were removed. Of these, 20 were assessed for full-text review, 3 quantitative studies were excluded, alongside 3 studies focusing primarily on sexual minority participants, meaning their findings were not relevant to TGD populations. A further eight studies were excluded due to insufficient focus on language and women’s healthcare. In total, six studies were eligible for inclusion. This is summarised in Figure 1, a PRISMA flowchart.

### Study characteristics

Characteristics of the six included studies are shown in Table 2.^22–27^

**Table 2.** Summary of characteristics.

Title	Author and date of publication	Journal source	Study type	Design and methods	Aims and contexts	Population	Study setting
Creating change with families: Reflections and recommendations for the care of gender diverse and LGBTQIA+ individuals and their families throughout pregnancy and birth.	Copeland et al., 2023	*Midwifery*	Qualitative case study.	Open-ended, semi structured, face–face interviews with thematic analysis.	To derive a deeper understanding of transgender and non-binary people’s experience of pregnancy and birth, and ways to modify practice to provide inclusive care.	Two transgender and gender diverse (TGD) participants	Tertiary hospital in South Australia
Stakeholder perceptions and experiences regarding access to contraception and abortion for transgender, non-binary, and gender-expansive individuals assigned female at birth in the U.S.	Fix et al., 2020	*Archives of Sexual Behaviour*	Qualitative descriptive study.	27 in-depth interviews with SRH stakeholders.	To collect diverse stakeholder perspectives on barriers and facilitators to contraception and abortion for TGD (referred to as transgender expansive, TGE, in study) recorded female at birth (referred to as assigned female at birth, AFAB, in study) individuals and identify recommendations for improving SRH services.	27 SRH stakeholders. 5 TGD individuals who had obtained contraception or abortion care and 22 clinicians, researchers and advocates experiences in TGD healthcare.	USA
Trans people’s experiences with assisted reproduction services: a qualitative study.	James-Abra et al., 2015	*Human Reproduction*	Qualitative descriptive study.	Secondary qualitative analysis of interview data from a community-based study focusing on SGMs.	To explore the experiences of trans individuals with assisted reproduction services and identify barriers and facilitators in accessing care.	9 TGD individuals and their partners.	Canada
Testing inclusive language revisions of the breastfeeding attrition prediction tool (BAPT) using cognitive interviewing: a pilot study.	Kinney et al., 2023	*Journal of Human Lactation*	Prospective qualitative study.	Exploratory, sequential qualitative study. Phase 1: completion of the revised gender inclusive BAPT. Phase 2: cognitive interviews assessing understanding and acceptance of the revised language.	To assess understanding and acceptance of an inclusive language revision of the BAPT.	16 “pregnant people.” 13 “females.” 1 TGD individual. 2 “not disclosed.” Aged between 18 and 44.	Vermont, USA
Sexual and gender minority patients’ first pelvic examination experiences: what clinicians need to know.	Ruiz et al., 2024	*Journal of Paediatric and Adolescent Gynaecology*	Cross-sectional qualitative study.	Qualitative cross-sectional study using purposive sampling and semi-structured interviews.	To identify factors that influence the first pelvic exam experiences of SGM adolescents and young adults who were recorded female at birth (referred to as AFAB in study.	14 (18–24-year-old) TGD (referred to as AFAB SGM in study) individuals who had had one previous pelvic exam.	Chicago, USA
Transgender and gender diverse individuals’ perspectives on discussions of foetal sex chromosomes in obstetrics care.	Tyrie et al., 2024	*Journal of Genetic Counselling.*	Qualitative sequential exploratory study.	Semi-structured video conference interviews followed by reflexive thematic analysis.	To explore TGD (referred to as GD in study) individuals’ opinions regarding foetal sex chromosome disclosure sessions	12 TGD individuals.	Canada and USA.

SRH: sexual and reproductive health; TGE: transgender expansive; AFAB: assigned female at birth; BAPT: Breastfeeding Attrition Prediction Tool; SGM: sexual and gender minority; GD: gender diverse.

All included studies were published in peer-reviewed journals between 2015 and 2024. All studies collected data through interviews. Geographically, three studies were based in the United States, one in both the United States and Canada, one in Canada, and one in Australia. There were 80 participants across the 6 studies: 43 TGD individuals, 22 stakeholders, 13 women who were not TGD, and 2 participants who did not disclose their gender identity. Four studies included TGD participants.^21,23,25,26^ Of the remaining two, one included TGD participants and stakeholders such as clinicians and researchers,^22^ while the other involved both women who did not identify as TGD and TGD participants.^24^ Overall, five of the studies focused on the views and experiences of TGD individuals, with only one article considering the perspectives of women who were not TGD (*n* = 13).

### Quality assessment

The outcomes of the CASP quality assessment varied across the included studies. A summary of the appraisal is provided in Table 3. All six studies clearly stated their research aims and appropriately employed qualitative methodology. However, only three studies adequately considered the relationship between the researcher and the participants^23–25^ and clearly stated their findings.^22,23,25^ This means that the results were presented transparently, supported by participant data, and clearly linked to the research question.

**Table 3.** CASP summary.

CASP questions	Copeland et al. (2023).	Fix et al. (2020).	James-Abra et al. (2015).	Kinney et al. (2023).	Ruiz et al. (2024).	Tyrie et al. (2024).
Was there a clear statement of the aims of the research?	Yes	Yes	Yes	Yes	Yes	Yes
Is a qualitative methodology appropriate?	Yes	Yes	Yes	Yes	Yes	Yes
Was the research design appropriate to address the aims of the research?	No	Can’t tell	Can’t tell	Yes	Yes	No
Was the recruitment strategy appropriate to the aims of the research?	Can’t tell	Yes	Yes	Yes	Yes	Can’t tell
Was the data collected in a way that addressed the research issue?	Yes	Can’t tell	Can’t tell	Yes	Yes	Yes
Has the relationship between researcher and participants been adequately considered?	No	No	Yes	Yes	Yes	No
Have ethical issues been taken into consideration?	Yes	Yes	Yes	Yes	Yes	No
Was the data analysis sufficiently rigorous?	No	Yes	Yes	Yes	Yes	Yes
Is there a clear statement of findings?	No	Yes	Yes	No	Yes	Can’t tell
Is the research valuable?	No	Yes	Yes	Yes	Yes	Yes

CASP: Critical Appraisal Skills Programme.

## Thematic analysis

### TGD experiences and perspectives

#### Language and communication

##### Desexed language

TGD study participants appreciated the incorporation of desexed language into women’s healthcare services, describing it as crucial for positive consultations.^21–26^ Terms such as “pregnant people” and “menstruating people” made them feel safer expressing their identities.^21,26^

However, views on the extent to which desexed language should be implemented – and approaches to its implementation – varied amongst TGD participants. Suggestions for implementation included asking all service users what their pronouns are during pregnancy care.^21^ Participants noted sexed terms like “mother” could feel exclusionary, creating “big wall(s)” in communication when receiving healthcare.^22^ Others emphasised the importance of inclusive, patient-specific terminology over blanket language reforms to better reflect diverse experiences.^22^ Heterogeneity in participants’ perspectives on desexed language implementation is clear, with some advocating for universal changes in communication for everyone.^21^ Others supported specific resources with language tailored to the TGD community.^22^

##### Importance of language and labelling

Two studies noted that healthcare professionals (HCPs) and healthcare institutions often used the terms sex, gender, and gender-identity interchangeably.^23,26^ Resources with “clear differentiation” between these terms were recommended to improve HCPs’ understanding of TGD individuals.^26^ However, participants varied in how they defined or understood these terms, with some linking sex, gender and gender identity, others not, and some undecided.

#### Education

##### Gaps in knowledge

Five studies highlighted a need for better clinical education regarding TGD healthcare.^21–23,25,26^ One participant expressed that inadequate education “can make otherwise amenable clinicians hesitant”^22^ with another recalling: clinicians would “get flustered” and refer to someone as “she” when they wish to be called “he” (misgender them). Misgendering is defined as interacting with someone in such a way that does not respect their gender identity such as using the incorrect pronouns (e.g., referring to a transman as “she”).^27^ Study participants linked misgendering with inexperience amongst clinicians. Enhanced education was seen as critical for fostering confidence in HCPs, improving patient relationships, and reducing fears surrounding misgendering.^21^

One TGD individual expressed embarrassment at clinicians’ attempts to avoid sexed terminology.^26^ They hoped: “as it gets more normalised it’ll be easier but not in an enforced – we have to change this right now and never use gender terms again – way.”

Copeland et al.,^21^ Fix et al.,^22^ and James-Abra et al.^23^ highlighted a need for education reform, both at university and hospital levels.

##### Basic training

Fix et al.^22^ identified HCPs’ unique perspectives in navigating desexed language implementation for their patients, recommending “introductory” and “beginner-level information.” It was reported that current systems, designed for women’s healthcare, are not suitable for TGD people. Therefore, it was suggested that changes should be introduced at a basic level to aid in understanding.

One HCP expressed the need for a non-judgemental learning environment, stating they should not feel “threatened, that they’re not competent or not able to be inclusive.”^22^ This highlights the importance of fostering supportive frameworks to aid HCPs in navigating language changes.

##### Education changes driven by TGD people

Recommendations for improving education included involving TGD individuals in curriculum development and teaching sessions to better reflect TGD individuals’ experiences.^22,23,26^ Participants expressed concerns about curricula designed by non-TGD individuals, with one stating, “they won’t get it right.”^22^ Collaboration between patients and HCPs was emphasised, as one participant noted, patients can “help HCPs understand. . . what you need.”^22^

#### Structural challenges

##### Clinical documentation

Difficulties regarding clinical documentation were identified.^21–26^ These included participants recalling editing patient forms to accommodate for their gender-identity, as forms did not allow entries such as “man wishing to conceive” in fertility clinics.^23^ There were also issues arising from documentation inadequately accommodating preferred names and pronouns.^22,23^ Links between clinical documentation and misgendering in consultations were postulated.^23^

Recommendations to alter documentation included tailoring language to each patient’s preferences, allowing individuals to select “their pronouns and preferred term to refer to human lactation from a drop-down menu.”^24^ One TGD participant expressed concerns that abrupt language changes, such as avoiding all sexed terms, may seem forced, but customising language for different groups could prevent exclusion.^26^

##### Institutional change

A lack of resources accommodating for TGD individuals was evident.^21,23,25^ This included patient-facing resources, such as leaflets and posters,^21^ and aspects of the physical environment such as sex-neutral toilets.^23^

#### Barriers to care

##### Fear and hesitation

Fear surrounding being misgendered in healthcare settings was identified across five studies.^21–23,25,26^ “You can expect to be misgendered the entire time you’re in the doctor’s office” recalled one participant.^21^ These studies highlighted that misgendering and fear of discrimination may deter some TGD individuals from accessing vital healthcare services, which may lead to cumulative negative health effects.

##### Vicious cycle

Prior negative experiences meant TGD individuals would “come in feeling really hesitant, like they are already going to be treated badly.”^23^ Fix et al.^22^ identified links between “insufficient provider knowledge, anticipated healthcare discrimination, gendered healthcare environments, and delays in care-seeking.”

### Experiences and perspectives of women who were not TGD

#### Language and communication

##### Communication barriers created by desexed language

Kinney et al.^24^ uniquely explored the perspectives of women who were not TGD on desexed language, including terms like “chest feeding.” While TGD participants in this study appreciated the changes, acceptance among most women who were not TGD was low. For example, some women described the terms as “odd,” “foreign,” and “unnatural” and one woman found that the term “body feeding” elicited traumatic memories of past sexual assault. Other findings included one participant reporting that they would not have completed the survey if “chest feeding” had been used. This could reflect wider issues, potentially damaging women’s trust and hindering both research participation and access to healthcare. Language which aims to promote inclusivity for TGD people may therefore inadvertently exclude or alienate other women.

##### Insufficient evidence on general population responses to desexed language

As mentioned, Kinney et al. was the only study to examine the acceptance and understanding of desexed language among women who were not TGD. The total sample size for this group was small (*n* = 13), and the specific focus on breastfeeding limited the types of desexed language explored. None of the included studies examined understanding of desexed terms amongst those with lower literacy skills or with limited English. Therefore, this review indicates that the extent to which desexed language is accepted and understood in the general population remains largely unexplored.

## Discussion

This systematic review explored the implications of desexed language in women’s healthcare, analysing six qualitative research studies.^21–26^ Thematic analysis yielded four overarching themes: language and communication, education, structural challenges, and barriers to care.

The themes are inextricably linked. Lack of training on TGD health and desexed language may result in communication difficulties with TGD patients. This can be exacerbated by structural challenges such as patient documents and sexed clinical environments. This creates barriers for TGD individuals seeking healthcare, potentially deterring access to services.

Desexed language aims to reduce barriers for the TGD population.^13^ Complexities arise as women’s healthcare must account for all female people including those who identify as TGD and, for example, women with low English literacy, that is, two vulnerable patient groups.^8,28^ Women’s healthcare services are still adapting to include TGD individuals, bringing significant uncertainty. Care must be taken to ensure that adaptations for TGD people do not worsen outcomes for the broader population of women.

Deficiencies in education were consistently found across the selected studies, contributing to clinicians’ difficulties with language modification. This was also identified in a survey study by Parameshwaran et al.^29^ who found a majority of 166 U.K. medical students reported deficient lesbian, gay, bisexual and transgender (LGBT) education and limited familiarity with gender terminology. The included studies recommended educational reform with input from TGD people. This was supported by Nowaskie and Patel,^30^ whose study of 940 U.S. medical students suggested that around 35 h of LGBT teaching improves knowledge. Practical approaches to education delivery were demonstrated by Thomspon et al.,^31^ whose role-play exercise, developed by TGD individuals and specialist HCPs, improved HCPs’ correct use of pronouns. However, Nowaskie and Patel’s recommendation to allocate 35 h of specific training may be unrealistic given broader educational pressures and well-documented gaps in women’s health issues, such as breastfeeding.^32^ Moreover, the study identified distinct health needs between lesbian, gay, and bisexual people versus TGD individuals, indicating combined teaching risks overlooking TGD-specific needs and the needs of lesbian and bisexual women.

HCPs in one included study emphasised a need for introductory level teaching.^22^ Clear distinctions between sex, gender, and gender-identity^33^ and how this determines patients’ preferred language could form the foundation for bettering relationships with TGD patients, a finding supported by Gribble et al.^11^ and all the studies in this review. One approach, outlined by Kaufman et al.,^34^ is to update patient documentation to include a field for “sex” and a separate, optional, section for “gender-identity” which can guide the appropriate use of pronouns. This supports the delivery of sex-specific care while ensuring TGD individuals are respected. The importance of this was illustrated by Stroumsa et al.,^35^ who detailed a case study involving a transgender man delivering a stillborn baby, where an incorrectly documented sex marker and staff misunderstanding that transgender men are female contributed to suboptimal care.

It is important to consider the perspectives of the broader population of women who are not TGD. Bartick et al.^8^ and Gribble et al.^10^ have argued that desexed language may reduce clarity and accessibility for many women, particularly those with lower literacy or limited English. Furthermore, there appears to be limited recognition that advocacy for desexed language centres the TGD population over the wider population. Kinney et al.^24^ was the only included study examining general understanding of desexed terms, finding low levels of acceptability among women who were not TGD. The remaining included studies considered the experiences and perspectives of TGD individuals, with one study interviewing HCPs.^22^ Given the risks of misinterpretation or rejection of gender-related questions, as observed in the U.K. 2021 census,^5^ it is important to assess broader population-level understanding and acceptability of all varieties of desexed language, including by those with lower English proficiency who may be more likely to mistakenly record a gender-identity different to their sex.^36^

Since our review was conducted, an additional study considering desexed language has been published. Nakijoba et al.^37^ examined the acceptance and understanding of desexed terminology across 5 ethnically diverse communities in Uganda with 146 participants. While conducted in a different cultural context, notable parallels with Kinney et al.’s^24^ perceptions of desexed language were found. Terms such as “a body with a vagina” were described as disrespectful and shameful by participants in Nakijoba et al.’s study. Other terms like “menstruating people” elicited confusion, with participants interpreting it as “someone who is always menstruating.” Nakijoba et al. discusses how desexed language implementation may be an “imposition of foreign values rather than a progressive step towards inclusivity,” echoing Gribble et al.’s^10^ discussion of “cultural imperialism.” These perspectives highlight the importance of culturally appropriate language, as well as the principles of clear plain language communication.^9^

Additionally, Ingram Market Research^38^ conducted a survey on 500 African American’s opinions on desexed language that was not included in our review as it is not peer-reviewed research. The majority of respondents favoured sexed terminology, with 93% preferred “mother” to “birthing person” and 94% preferred “breastfeeding” to “chest feeding.” Notably, 64% reported they would be much less or less likely to trust medical professionals who employ desexed language, and 48% reported being much less or less comfortable with HCPs introducing themselves with their pronouns or displaying pronouns on name badges. These findings regarding “trust” are mirrored in Kinney et al.’s study, where one participant reported they would not partake in surveys using desexed language.

Taken together, the findings of Nakijoba et al., Kinney et al., and Ingram Market Research indicate significant concerns regarding the acceptability of implementing desexed language. There are also significant gaps in current research, particularly with regard to women who are not TGD. Only one study in our review examined understanding of desexed terms in this population, and no studies considered participants with lower literacy or limited English proficiency. Research outside Western English-speaking contexts is also limited. In addition, although this review found that TGD people overwhelmingly accept and support the use of desexed language, there is no research examining how desexed language affects comprehension for this group.

Gribble^17^ discussed various factors contributing to limited research surrounding desexed language implementation. The controversial nature of the topic was discussed, noting her personal experience of attacks and deplatforming following her publications. Gribble et al. postulated that some research may not have reached publication because the findings were not aligned with prevailing expectations, and funders, such as government-funded research institutes, may be reluctant to support studies in this area.

### Other limitations with the literature

This systematic review provides a thorough, systematic overview of the qualitative evidence regarding the implications of desexed language in women’s healthcare and healthcare literature using a comprehensive search strategy. However, there are some limitations of the included evidence. Copeland et al.^21^ based recommendations, such as asking all patients their pronouns, on a sample size of only two. Of the six studies in this review, only one^24^ considered the impact of desexed language revisions on women. The predominance of TGD-only populations in several studies may have influenced the findings.^21,23,25^ The majority (*n* = 4) of articles^21,23,25,26^ exclusively considered the TGD experience. The 2021 Census of England and Wales estimated the TGD population at 0.5%,^5^ and only a subset of this group will be recorded female at birth and subsequently access women’s healthcare services. For context, it has been estimated by Stroumsa et al.^39^ that approximately 0.07% of U.S. births occur among TGD people. Although small in number, this does not diminish the importance of this group receiving appropriate care.

Four studies did not consider the relationship between the researcher and the participants. Two studies reported research teams composed entirely of TGD individuals, whereas one study reported partial inclusion of TGD individuals within the research team. This could be a strength as it offers a team with experience who may understand some of the issues faced, but it could also have influenced interview techniques and data analysis. In one study, interviews were conducted by participants’ own midwives, a relationship not acknowledged by the authors, which could have limited participants’ willingness to provide open and honest answers if they felt this could influence their care. Another potential limitation is the sensitive nature of the subject matter which might have led participants to provide socially desirable responses. The authors of this systematic review are four women who are not TGD, which may also have influenced analysis and interpretation of findings.

Despite these limitations, this review is the first to examine the qualitative evidence base on the implementation of desexed language in women’s healthcare services and identifies important findings how such language is used in these settings.

## Conclusion

This review examined the implications of desexed language in women’s healthcare. Regarding TGD people, it has revealed the importance of appropriate language and communication, the need for improved education for HCPs, structural challenges and barriers to care. Development of specific resources, tailored to the TGD population, could accommodate their health needs while preserving sexed terminology in women’s healthcare. Regarding women who are not TGD, the limited evidence available suggests that desexed language may undermine clear communication, is poorly accepted and risks obscuring women’s health needs. There is a need for further research on the implications of desexed language for the broader population of women as well as TGD people. Without such evidence, broad implementation of desexed language cannot be considered an evidence-based intervention.

## Supplemental Material

sj-pdf-1-whe-10.1177_17455057261430199 – Supplemental material for A qualitative systematic review of the implications of desexed language in women’s healthcare and healthcare literature Supplemental material, sj-pdf-1-whe-10.1177_17455057261430199 for A qualitative systematic review of the implications of desexed language in women’s healthcare and healthcare literature by Abigail Greenfield, Ciara Higley, Naomi Black and Majel McGranahan in Women's Health

## Appendix A: Medline search string

### Search terms for language

“Non-specific sex language” “Gender-inclusive language” “Gender-sensitive language” “Gender-affirming language” “Non-binary language” “Transgender-inclusive language” “De-sexed language”

“Non-gendered terminology” “Sex-neutral language” “LGBTQ* inclusive language” “Queer-affirming language” “Gender-neutral pronouns” “Non-binary inclusive” “Gender diversity”

“Trans-inclusive communication” “Affirming language”

“Respectful language” “Cisgender-inclusive language” “Intersectional language” “Diverse gender representation” “Sexual and gender minority”

“Transgender patient communication” “Gender neutral language”

“Inclusive terminology” “Birth givers”

“Birthing individuals” “Cervix havers” “Menstruators” “Chest-feeders”

“People with uteruses” “Pregnant individuals”

### Search terms for women’s health

“Women’s health”

“Female reproductive health”

“Gynaecology”

“Gynecology”

“Gynaecological care”

“Obstetrics and gynaecology”

“Breast health”

“Cervical cancer screening” “Abortion”

“Contraception” “Fertility”

“Menopausal care” “Prenatal care” “Postnatal care” “Ovarian health” “Uterine health” “Mammography” “Pap smear” “Pregnancy” “Reproduction” “Maternity”

“Pelvic health” “Sexual health” “Endometriosis”

“Polycystic ovary syndrome” “PCOS”

“Preconception care” “Miscarriage” “Gynaecological surgeries” “Postpartum depression” “Breastfeeding”

“Vaginal health” “Hysterectomy” “Tubal ligation” “IVF”

“In vitro fertilization” “Gestational diabetes” “Perimenopausal care” “Pelvic organ prolapse” “Uterine fibroids” “Endometrial cancer” “Reproductive rights” “Menstrual health”
